# 582 Expanding Burn Expertise Through Telemedicine for the Frontliners during the COVID-19 Pandemic

**DOI:** 10.1093/jbcr/irac012.210

**Published:** 2022-03-23

**Authors:** Myra Alexandra P Firaza, Maria Redencion B Abella

**Affiliations:** Jose R. Reyes Memorial Medical Center, Manila, National Capital Region; Jose R. Reyes Memorial Medical Center, Cainta, Rizal

## Abstract

**Introduction:**

Inexperience of frontliners and referring physicians from non-specialty centers in burn wound assessment results to the incorrect triage of patients, thereby aggravating the current hospital situations and causing unnecessary exposures. Emergency care in burn centers in developing countries must strike a balance between doctor and patient safety, and uncompromised care of burn patients. Telemedicine is deemed a valid and sound option to maintain social distancing and promote safety, yet provide proper burn care. It is a valuable and indispensable tool for all doctors of all branches of medicine and surgery. Although many of its limitations in developing countries are still being unraveled, the benefits of this technology are being realized worldwide. This study determined the accuracy and timeliness in diagnosing and classifying burn patients assessed by a frontliner non-burn specialist in-person (NBSP), a Burn Specialist online (BSO), and a Burn Specialist in-person (BSP).

**Methods:**

All burn patients (January to March 2021) with signed consent for participation were photographed in a standardized manner by the NBSP and referred to a BSO via an online messaging application. These patients were also assessed independently by the BSP. The % total body surface area (TBSA), burn depth classification, and the time the patients were seen by the NBSP, the time the online referrals were sent to the BSO through the messaging application, the time the BSO sent the diagnoses, and the time of assessment by the BSP, were recorded. One-Way Repeated Measures Analysis of Variance (ANOVA) with and without blocking were done.Post-hoc Tukey-Test was used to analyze the pair-wise differences for any ANOVA that showed significant statistical differences.

**Results:**

Data gathered from 82 patients throughout the 3-month study duration demonstrated that burn size (% TBSA) among the three different physicians (NBSP, BSO, BSP) was not statistically significant (p=0.8794). Our analysis also showed no statistical difference for the 19 different body parts per patient and burn depth classification (p=0.9718). One-way ANOVA tests on timeliness were statistically significant with a p-value of p< 0.0001. A post-hoc comparison using Tukey test revealed no statistical significance between the BSO and BSP (p=0.892).

**Conclusions:**

Smartphone telemedicine platform through photographic transfer and analysis is an accurate method in estimating burn size and depth classification. Timeliness can be improved with a dedicated 24/7 online available burn specialists and a reliable network access. Hence, frontliners can refer to burn specialists in a developing country using this telemedicine platform for optimum burn care with an accurate diagnosis and overcome the challenges during and even after this pandemic.

